# Genome-wide association study reveals novel loci associated with body size and carcass yields in Pekin ducks

**DOI:** 10.1186/s12864-018-5379-1

**Published:** 2019-01-03

**Authors:** Meng-Ting Deng, Feng Zhu, Yu-Ze Yang, Fang-Xi Yang, Jin-ping Hao, Si-Rui Chen, Zhuo-Cheng Hou

**Affiliations:** 1 National Engineering Laboratory for Animal Breeding and MARA Key Laboratory of Animal Genetics and Breeding, Department of Animal Genetics and Breeding, China Agricultural University, Beijing, 100193 China; 2 Beijing General Station of Animal Husbandry, Beijing, 100107 China; 3 Beijing Golden Star Duck Co., LTD, Beijing, 100076 China

**Keywords:** Carcass trait, Body size, Genome-wide association study, Pekin duck

## Abstract

**Background:**

Pekin duck products have become popular in Asia over recent decades and account for an increasing market share. However, the genetic mechanisms affecting carcass growth in Pekin ducks remain unknown. This study aimed to identify quantitative trait loci affecting body size and carcass yields in Pekin ducks.

**Results:**

We measured 18 carcass traits in 639 Pekin ducks and performed genotyping using genotyping-by-sequencing (GBS). Loci-based association analysis detected 37 significant loci for the 17 traits. Thirty-seven identified candidate genes were involved in many biological processes. One single nucleotide polymorphism (SNP) (Chr1_140105435 A > T) located in the intron of the ATPase phospholipid transporting 11A gene (*ATP11A*) attained genome-wide significance associated with five weight traits. Eight SNPs were significantly associated with three body size traits, including the candidate gene plexin domain containing 2 (*PLXDC2*) associated with breast width and tensin 3 (*TNS3*) associated with fossil bone length. Only two SNPs were significantly associated with foot weight and four SNPs were significantly associated with heart weight. In the gene-based analysis, three genes (*LOC101791418*, *TUBGCP3* (encoding tubulin gamma complex-associated protein 3), and *ATP11A*) were associated with four traits (42-day body weight, eviscerated weight, half-eviscerated weight, and leg muscle weight percentage). However, no loci were significantly associated with leg muscle weight in this study.

**Conclusions:**

The novel results of this study improve our understanding of the genetic mechanisms regulating body growth in ducks and thus provide a genetic basis for breeding programs aimed at maximizing the economic potential of Pekin ducks.

**Electronic supplementary material:**

The online version of this article (10.1186/s12864-018-5379-1) contains supplementary material, which is available to authorized users.

## Background

China is the world’s largest producer and consumer of domestic ducks, with Chinese duck production accounting for 75% of duck production worldwide (FAO). Furthermore, duck farming has been growing rapidly in China, and in addition to breast meat and eggs, secondary products, such as duck neck and wing are also very popular. However, these secondary products including the head, neck, feet, and wings are more expensive than breast muscle, and it is therefore necessary to pay more attention to the traits affecting these secondary products.

Previous breeding programs have not focused on the composition of these other body parts in ducks. However, molecular-based breeding programs now have been applied in animal breeding, and numerous quantitative trait loci (QTL) affecting carcass traits have been identified in various animals. Several biological pathways related to carcass traits in cattle have been identified, including peroxisome proliferator-activated receptor signaling, while some significant associations were detected in close proximity to genes with known roles in animal growth, such as glucagon and leptin [1–3]. In pigs, the number of copies of the vertebrae development associated gene (*VRTN*) was related to body length [4], while SSC1 and SSC8 include several loci related to carcass traits in pigs. Three genes, *TBC1D1* (encoding TBC1 domain family member 1), *BAAT* (bile acid-CoA: amino acid N-acyltransferase), and *PHLPP1* (PH domain and Leucine-rich repeat protein phosphatase 1) were highlighted as functionally plausible candidate genes for pig growth and fatness traits [5, 6]. In addition to pigs and cattle, similar studies have been conducted in poultry, and numerous candidate genes associated with carcass traits have been found in chickens, including *TBC1D1*, *LCORL* (ligand-dependent nuclear receptor corepressor-like), *LAP3* (leucine aminopeptidase 3), *LDB2* (LIM-domain-binding 2), and *TAPT1* (transmembrane anterior posterior transformation 1) [7–10]. However, compared with other domestic animals, study about the genetic basis of growth traits in ducks is lacking. Previous studies suggested that the fat mass and obesity-associated gene (*FTO*) [11] and mutations in intron 2 of the growth hormone gene (*GH*) [12] influenced duck carcass and meat quality traits, while Zhang et al. [13] also showed that the perilin gene (*PLIN*) affected duck carcass and fat traits.

The current chicken QTL database [14], includes more than 1500 QTLs related to body weight traits, but QTLs for traits related to the composition of subordinate body parts, such as neck length, fossil bone length, and foot weight, are still rare. Furthermore, few studies have been conducted in ducks, and no animal QTL database currently includes duck-related QTL. This lack of information needs to be addressed to support the development of duck breeding projects.

In the present study, we conducted a genome-wide association study (GWAS) in 639 Pekin ducks using genotyping-by-sequencing (GBS) [15]. We measured or derived a total of 18 body size and carcass yields traits, and aimed to identify potential loci and candidate genes affecting these traits. To our knowledge, this is the first large-scale GWAS investigation of duck carcass traits. These QTL information will not only facilitate the study of molecular genetic mechanisms, but may also improve the accuracy of genetic selection for carcass traits in ducks.

## Results

### Descriptive statistics

Mean values (and standard deviations) for body size and carcass yields traits are shown in Table 1 and the phenotypic correlations are shown in Table 2. The highest phenotypic correlation (0.98) was between DW and EW, and the lowest correlation (− 0.24) was between DP and LMWP. The phenotypic correlations among the four body weight traits (DW, EW, HEW, and BW42) were all > 0.90, while the correlations between leg muscle percentage and the other 17 traits were all < 0.2, and were mostly negative.

**Table 1:** Descriptive statistics of phenotypic data

Trait type	Trait	Mean ± S.D.	Max	Min
Body size	Neck length (NL, cm)	20.3 ± 1.2	23.5	17.5
	Fossil bone length (FBL, cm)	14.1 ± 0.7	16	12
	Breast width (BrW, cm)	10.7 ± 0.5	12.6	9.3
Carcass yield	Dressed weight (DW, kg)	2.7 ± 0 .2	3.4	2.1
	Dressed percentage (DP, %)	87.1 ± 1.8	92.6	83.2
	Eviscerated weight (EW, kg)	2.3 ± 0.2	2.8	1.7
	Eviscerated weight percentage (EWP, %)	73.4 ± 1.9	90.5	63.1
	Half-eviscerated weight (HEW, kg)	2.4 ± 0.2	3.0	1.8
	Percentage of half-eviscerated yield (HEWP, %)	77.9 ± 1.9	96.0	66.7
	42-day body weight (BW42, kg)	3.1 ± 0.3	3.9	2.4
Internal organs	Heart weight (HW, g)	17 ± 2.8	28	9.5
	Liver weight (LW, g)	69.5 ± 9.7	120.6	42.6
Cut	Foot weight (FW, g)	66 ± 7.3	91.6	45.5
	Wing weight (WW, g)	111.7 ± 9.7	141.8	81
	Breast muscle weight (BMW, g)	238.2 ± 36.8	399	134
	Breast muscle weight percentage (BMWP, %)	10.5 ± 1.2	16.4	7.1
	Leg muscle weight (LMW, kg)	234 ± 86.9	456	123
	Leg muscle weight percentage (LMWP, %)	10.4 ± 3.8	37.3	11.7

**Table 2:** heritability (bold,diagonal), genetic correlations (above diagonal) and phenotypic correlations (below diagonal) for body size and carcass traits (±standard errors)

Trait	NL	FBL	BrW	DW	DP	EW	EWP	HEW	HEWP	BW42	HW	LW	FW	WW	BMW	BMWP	LMW	LMWP
NL	>0.42 ± 0.14	>0.31 ± 0.17	>0.11 ± 0.24	>0.42 ± 0.14	>0.18 ± 0.33	>0.38 ± 0.15	>− 0.07 ± 0.21	>0.39 ± 0.15	>0.04 ± 0.20	>0.41 ± 0.14	>0.31 ± 0.21	>0.45 ± 0.14	>0.52 ± 0.13	>0.20 ± 0.16	>0.13 ± 0.17	>−0.14 ± 0.21	>0.14 ± 0.24	>−0.10 ± 0.25
FBL	0.40	0.45 ± 0.14	0.48 ± 0.15	0.81 ± 0.10	0.44 ± 0.31	0.76 ± 0.11	−0.12 ± 0.20	0.75 ± 0.11	>−0.15 ± 0.19	0.81 ± 0.10	0.83 ± 0.16	0.67 ± 0.15	0.68 ± 0.13	0.86 ± 0.12	0.68 ± 0.14	0.44 ± 0.20	0.37 ± 0.22	0.11 ± 0.24
BrW	0.11	0.44	0.29 ± 0.11	0.49 ± 0.18	0.41 ± 0.37	1.00 ± 0.17	0.04 ± 0.25	1.00 ± 0.16	−0.03 ± 0.24	1.00 ± 0.25	0.98 ± 0.18	0.77 ± 0.26	0.73 ± 0.19	1.00 ± 0.20	1.00 ± 0.12	0.78 ± 0.20	0.20 ± 0.28	−0.12 ± 0.29
DW	0.28	0.57	0.54	0.60 ± 0.13	0.48 ± 0.24	0.97 ± 0.01	0.10 ± 0.16	0.97 ± 0.01	0.13 ± 0.16	0.99 ± 0.01	1.00 ± 0.10	1.00 ± 0.07	0.79 ± 0.07	0.85 ± 0.06	0.75 ± 0.08	0.34 ± 0.17	0.33 ± 0.18	0.07 ± 0.20
DP	0.11	0.12	−0.02	0.34	0.46 ± 0.12	0.49 ± 0.25	0.38 ± 0.25	0.50 ± 0.25	0.41 ± 0.24	0.39 ± 0.28	0.70 ± 0.30	0.39 ± 0.30	0.53 ± 0.28	0.18 ± 0.29	0.50 ± 0.30	0.38 ± 0.39	0.82 ± 0.49	0.55 ± 0.47
EW	0.27	0.57	0.54	0.98	0.32	0.58 ± 0.13	0.30 ± 0.15	1.00 ± 0.25	0.32 ± 0.15	0.96 ± 0.01	1.00 ± 0.10	0.97 ± 0.09	0.82 ± 0.07	0.88 ± 0.06	0.80 ± 0.08	0.39 ± 0.17	0.36 ± 0.19	0.09 ± 0.20
EWP	0.04	0.11	−0.01	0.23	0.74	0.35	0.44 ± 0.16	0.28 ± 0.15	0.99 ± 0.01	0.02 ± 017	0.09 ± 0.22	0.01 ± 0.17	0.29 ± 0.17	0.15 ± 0.17	0.31 ± 0.16	0.29 ± 0.21	0.50 ± 0.28	0.45 ± 0.29
HEW	0.26	0.56	0.54	0.98	0.31	1.00	0.32	0.58 ± 0.13	0.31 ± 0.15	0.97 ± 0.01	1.00 ± 0.10	0.98 ± 0.08	0.81 ± 0.07	0.88 ± 0.06	0.78 ± 0.08	0.36 ± 0.17	0.34 ± 0.19	0.07 ± 0.20
HEWP	0.02	0.08	−0.01	0.28	0.75	0.38	0.97	0.37	0.43 ± 0.16	0.05 ± 0.16	0.07 ± 0.22	0.08 ± 0.17	0.28 ± 0.17	0.12 ± 0.17	0.25 ± 0.16	0.16 ± 0.21	0.41 ± 0.28	0.35 ± 0.28
BW42	0.27	0.57	0.58	0.97	0.11	0.95	0.06	0.96	0.10	0.64 ± 0.13	0.98 ± 0.10	1.00 ± 0.07	0.77 ± 0.07	0.88 ± 0.06	0.74 ± 0.09	0.34 ± 0.17	0.30 ± 0.18	0.04 ± 0.20
HW	0.11	0.31	0.28	0.52	0.27	0.50	0.19	0.51	0.23	0.48	0.34 ± 0.13	0.95 ± 0.16	0.88 ± 0.12	0.77 ± 0.15	0.91 ± 0.15	0.45 ± 0.23	0.55 ± 0.20	0.26 ± 0.25
LW	0.26	0.37	0.32	0.66	0.09	0.63	−0.03	0.65	0.08	0.68	0.36	0.60 ± 0.13	0.81 ± 0.09	0.86 ± 0.12	0.54 ± 0.15	0.09 ± 0.19	0.16 ± 0.16	−0.01 ± 0.20
FW	0.42	0.50	0.37	0.70	0.14	0.71	0.14	0.70	0.13	0.71	0.45	0.58	0.55 ± 0.13	0.87 ± 0.06	0.58 ± 0.14	0.24 ± 0.20	0.36 ± 0.19	0.13 ± 0.21
WW	0.33	0.54	0.42	0.74	0.20	0.75	0.25	0.73	0.23	0.72	0.42	0.46	0.77	0.57 ± 0.13	0.64 ± 0.12	0.28 ± 0.18	0.38 ± 0.20	0.14 ± 0.21
BMW	0.00	0.42	0.44	0.65	0.22	0.67	0.28	0.66	0.28	0.63	0.42	0.29	0.34	0.46	0.63 ± 0.14	0.86 ± 0.05	0.56 ± 0.18	0.34 ± 0.19
BMWP	−0.19	0.15	0.19	0.15	0.06	0.17	0.13	0.16	0.10	0.15	0.19	−0.07	−0.06	0.07	0.84	0.40 ± 0.15	0.67 ± 0.25	0.50 ± 0.23
LMW	0.05	0.19	0.11	0.17	−0.16	0.21	−0.01	0.20	−0.03	0.23	0.29	0.07	0.29	0.22	0.15	0.04	0.30 ± 0.15	0.96 ± 0.02
LMWP	−0.01	0.08	−0.01	− 0.05	−0.24	− 0.02	−0.09	− 0.02	−0.12	0.01	0.19	−0.07	0.14	0.05	−0.01	0.00	0.97	0.91 ± 0.15

Neck length (NL), Fossil bone length (FBL), Breast width (BrW), Dressed weight (DW), Dressed percentage (DP), Eviscerated weight (EW), Eviscerated weight percentage (EWP), Half-eviscerated weight (HEW), Percentage of half-eviscerated yield (HEWP), 42-day body weight (BW42), Heart weight (HW), Liver weight (LW), Foot weight (FW), Wing weight (WW), Breast muscle weight (BMW), Breast muscle weight percentage (BMWP), Leg muscle weight (LMW), Leg muscle weight percentage (LMWP)

### Genetic parameters

The genetic parameters of all traits are shown in Table 2. The estimated heritability of these 18 traits ranged from 0.30–0.91. LMWP showed the highest heritability, suggesting a considerable genetic contribution to carcass yields and body size. There was also a high genetic correlation between FBL and body weight traits, with correlation coefficients > 0.75, and a high genetic correlation between body weight and internal organ weight, with a correlation coefficient > 0.90.

### Loci-based analysis

**Fig. 1:**
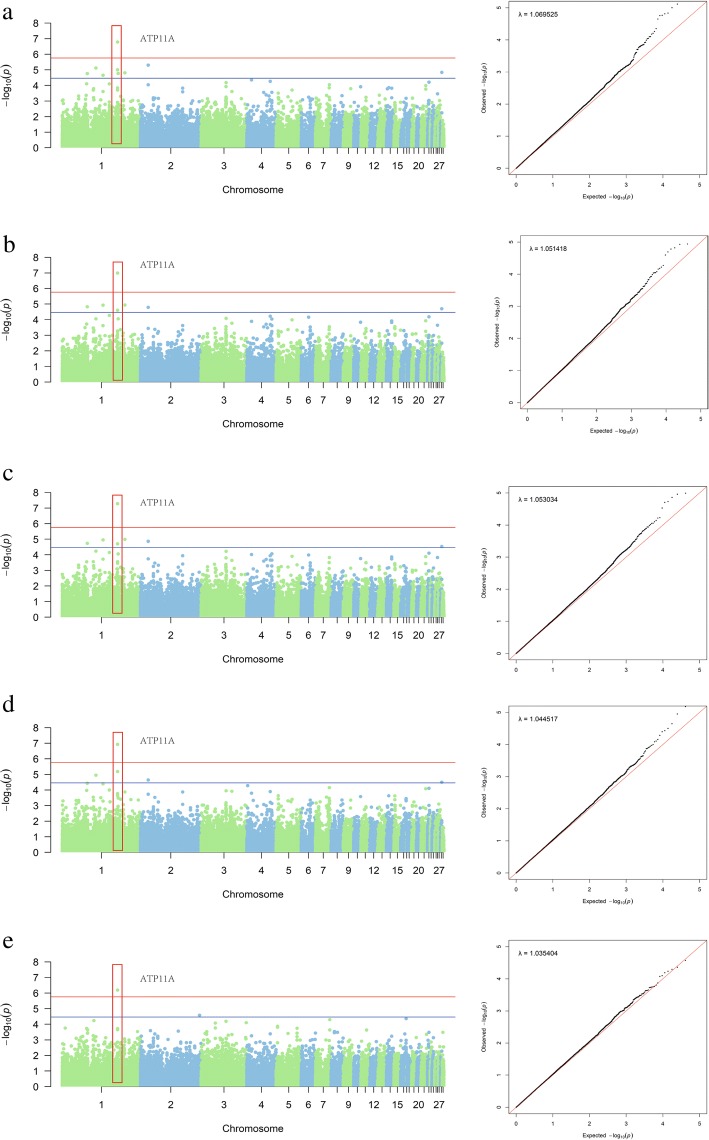
Manhattan plots showing associations of all SNPs with five traits and quantile-quantile (Q-Q) plots of the GLM (black dots) for five traits (a. DW, b. EW, c. HEW, d. BW42, e. WW). One SNP reached genome-wide significance for the five above traits. In Manhattan plots, SNPs are plotted on the x-axis according to their position on each chromosome, against association with these traits on the y*-*axis (shown as −log_10_*P*-value). Blue solid line indicates suggestive significance association (*P* = 3.48E− 05), and red solid line shows genome-wide significance with a *P*-value threshold of 1.74E− 06. In Q-Q plots, expected *P*-values under the null hypothesis are plotted on the x-axis and observed *P*-values on the y-axis. The estimated genomic inflation factor λ ranged from 1.03–1.06

A total of 37 significant QTLs (*P* < 3.48E− 05) across 14 chromosomes were identified by loci-based analysis (Table 3; Additional file 1: Figure S1). The estimated genomic inflation factor λ ranged from 1.01–1.06, suggesting no population stratification in the studied population (Additional file 1: Figure S2). One result indicated a genome-wide significant QTL (SNP Chr1_140105435 A > T) (Fig. 1).

**Table 3:** Information for all significant SNP related to body size and carcass traits

Trait	Nb_snp_	Chr	Position (ps)	AF	Beta	*p*-value	Var (%)	Candidate gene	Distance
BMW	2	3	11,176,648	0.276	−2.55E-01	2.53E-05	4.1	*TMEM17*	7442 bp upstream
		10	1,656,050	0.366	−2.35E-01	2.96E-05	4.1	*LOC101804888*	11,627 bp downstream
BMWP	3	1	867,910	0.45	2.56E-01	3.18E-06	8.1	*LOC101803092*	within
		1	47,950,429	0.397	−2.39E-01	6.71E-06	6.9	*ATP2B1*	107,815 bp upstream
		1	47,950,615	0.402	−2.31E-01	1.82E-05	6.4	*ATP2B1*	107,629 bp upstream
BrW	4	2	19,666,206	0.479	−2.39E-01	1.45E-05	9.8	*PLXDC2*	5029 bp downstream
		2	24,791,499	0.362	−2.60E-01	7.69E-06	10.8	*LOC101799835*	within
		6	31,223,243	0.369	2.22E-01	2.14E-05	7.9	*WDR11*	29,320 bp upstream
		21	1,548,509	0.427	2.31E-01	2.59E-05	9	*EYA2*	28,420 bp downstream
BW42	5	1	85,234,035	0.455	−2.29E-01	1.12E-05	4.1	*LOC101789880*	within
		1	140,105,435	0.305	−2.64E-01	1.20E-07	4.6	*ATP11A*	within
		1	140,105,568	0.47	2.16E-01	6.54E-06	3.6	*ATP11A*	within
		2	19,666,336	0.229	−2.29E-01	2.25E-05	2.9	*PLXDC2*	5159 bp downstream
		28	3,372,077	0.024	−6.23E-01	3.16E-05	2.8	*LOC101803004*	1892 bp downstream
DP	2	1	7,468,875	0.358	2.22E-01	1.07E-05	4.9	*GRM3*	121,336 bp downstream
		6	33,060,881	0.368	2.35E-01	3.09E-06	5.6	*CTBP2*	30,880 bp downstream
DW	8	1	63,206,125	0.395	−2.41E-01	1.75E-05	4.6	*CACNA1C*	within
		1	85,234,035	0.455	−2.28E-01	7.68E-06	4.3	*LOC101789880*	within
		1	140,105,435	0.305	−2.55E-01	1.65E-07	4.6	*ATP11A*	within
		1	140,105,568	0.47	2.06E-01	9.97E-06	3.5	*ATP11A*	within
		1	141,660,288	0.44	2.05E-01	1.72E-05	3.4	*LOC101802568*	26,100 bp downstream
		1	158,535,990	0.35	−2.14E-01	1.54E-05	3.5	*KLF5*	44,712 bp upstream
		2	19,666,336	0.229	−2.40E-01	5.01E-06	3.4	*PLXDC2*	5159 bp downstream
		28	3,372,077	0.024	−6.41E-01	1.46E-05	3.2	*LOC101803004*	1892 bp downstream
EW	6	1	63,206,125	0.395	−2.47E-01	1.51E-05	5	*CACNA1C*	within
		1	140,105,435	0.305	−2.65E-01	1.02E-07	5.1	*ATP11A*	within
		1	140,105,568	0.47	2.02E-01	2.52E-05	3.5	*ATP11A*	within
		1	158,535,990	0.35	−2.22E-01	1.15E-05	3.9	*KLF5*	44,712 bp upstream
		2	19,666,336	0.229	−2.32E-01	1.65E-05	3.3	*PLXDC2*	5159 bp downstream
		28	3,372,077	0.024	−6.37E-01	2.02E-05	3.3	*LOC101803004*	1892 bp downstream
EWP	4	2	35,813,875	0.372	−2.27E-01	2.74E-05	5.5	*CHN2*	within
		3	89,970,339	0.313	−2.24E-01	2.59E-05	4.9	*LGSN*	57,852 bp downstream
		13	17,512,359	0.391	−2.18E-01	1.02E-05	5.1	*FOXP1*	2160 bp downstream
		18	5,703,171	0.433	2.29E-01	8.74E-06	5.8	*PPP1R26*	37,536 bp upstream
FBL	3	2	40,157,075	0.49	2.44E-01	1.50E-05	6.6	*OXSM*	15,327 bp downstream
		2	52,398,515	0.419	2.40E-01	1.13E-05	6.2	*TNS3*	within
		2	137,295,017	0.372	−2.18E-01	3.17E-05	5	*TMEM74*	1377 bp upstream
FW	2	1	148,339,868	0.018	7.08E-01	7.73E-06	3.2	*ABCC4*	within
		4	59,568,289	0.02	6.09E-01	1.62E-05	2.6	*LDB2*	17,964 bp downstream
HEW	6	1	63,206,125	0.395	−2.46E-01	1.85E-05	5	*CACNA1C*	within
		1	140,105,435	0.305	−2.72E-01	5.27E-08	5.4	*ATP11A*	within
		1	140,105,568	0.47	2.06E-01	1.98E-05	3.6	*ATP11A*	within
		1	158,535,990	0.35	−2.24E-01	1.02E-05	3.9	*KLF5*	44,712 bp upstream
		2	19,666,336	0.229	−2.35E-01	1.38E-05	3.4	*PLXDC2*	5159 bp downstream
		28	3,372,077	0.024	−6.28E-01	2.95E-05	3.2	*LOC101803004*	1892 bp downstream
HEWP	2	1	196,705,092	0.357	2.18E-01	2.95E-05	5.1	*LOC101797437*	5799 bp downstream
		13	17,512,359	0.391	−2.12E-01	2.27E-05	5	*FOXP1*	2160 bp downstream
HW	4	1	158,535,990	0.35	−2.53E-01	2.06E-06	8.6	*KLF5*	44,712 bp upstream
		3	64,428,149	0.434	2.29E-01	8.58E-06	7.6	*GJA1*	14,583 bp downstream
		5	22,865,850	0.351	2.30E-01	6.43E-06	7.1	*AQR*	11,077 bp upstream
		7	11,397,351	0.312	−2.35E-01	2.62E-05	7	*PCNT*	within
LW	5	1	119,976,059	0.407	−2.12E-01	1.31E-05	3.6	*LOC101803212*	13,772 bp downstream
		1	34,185,127	0.242	−2.24E-01	2.47E-05	3.1	*PPM1H*	within
		4	427,963	0.473	2.19E-01	9.55E-06	4	*LOC101798797*	148,591 bp downstream
		4	59,555,379	0.016	6.79E-01	3.37E-05	2.4	*LDB2*	4954 bp downstream
		8	23,698,948	0.419	2.21E-01	2.95E-05	4	*AGBL4*	within
LMWP	1	20	2,198,171	0.385	−2.40E-01	2.73E-05	3	*AUTS2*	30,531 bp downstream
NL	1	1	130,709,943	0.354	−2.01E-01	2.79E-05	4.4	*XG*	within
WW	2	1	140,105,435	0.305	−2.44E-01	6.49E-07	4.4	*ATP11A*	within
		2	149,672,484	0.015	8.53E-01	2.67E-05	3.8	*FAM135B*	33,375 bp downstream

Neck length (NL), Fossil bone length (FBL), Breast width (BrW), Dressed weight (DW), Dressed percentage (DP), Eviscerated weight (EW), Eviscerated weight percentage (EWP), Half-eviscerated weight (HEW), Percentage of half-eviscerated yield (HEWP), 42-day body weight (BW42), Heart weight (HW), Liver weight (LW), Foot weight (FW), Wing weight (WW), Breast muscle weight (BMW), Breast muscle weight percentage (BMWP), Leg muscle weight (LMW), Leg muscle weight percentage (LMWP). Chr, chromosomeID; Nb_snp_, number of significant SNP, retaining the most significant one if the distance between multiple SNP of the same trait is less than 0.15 Mb; AF, minor allele frequency; Beta, the estimate coefficient; Var (%), % of genetic variance explained by the top SNP

Eight non-overlapping QTLs were obtained for body size traits. The most significant of these (Chr2_24791499_A > T; located in the intron between exons 8 and 9 of *LOC101799835*) was related to BrW (*P* = 7.67E− 06) and accounted for 10.8% of the genetic variance. A SNP on chromosome 1 (Chr1 130,709,943 A > T; located in the intron between exons 8 and 9 of the Xg blood group gene, *XG*) was the only locus significantly associated with NL. Three significant QTLs in separate regions of Chr2 were associated with FBL, with the most significant (SNP chr2_52398515_C > T; *P* = 1.13E− 05) accounting for 6.2% of the genetic variance.

**Fig. 2:**
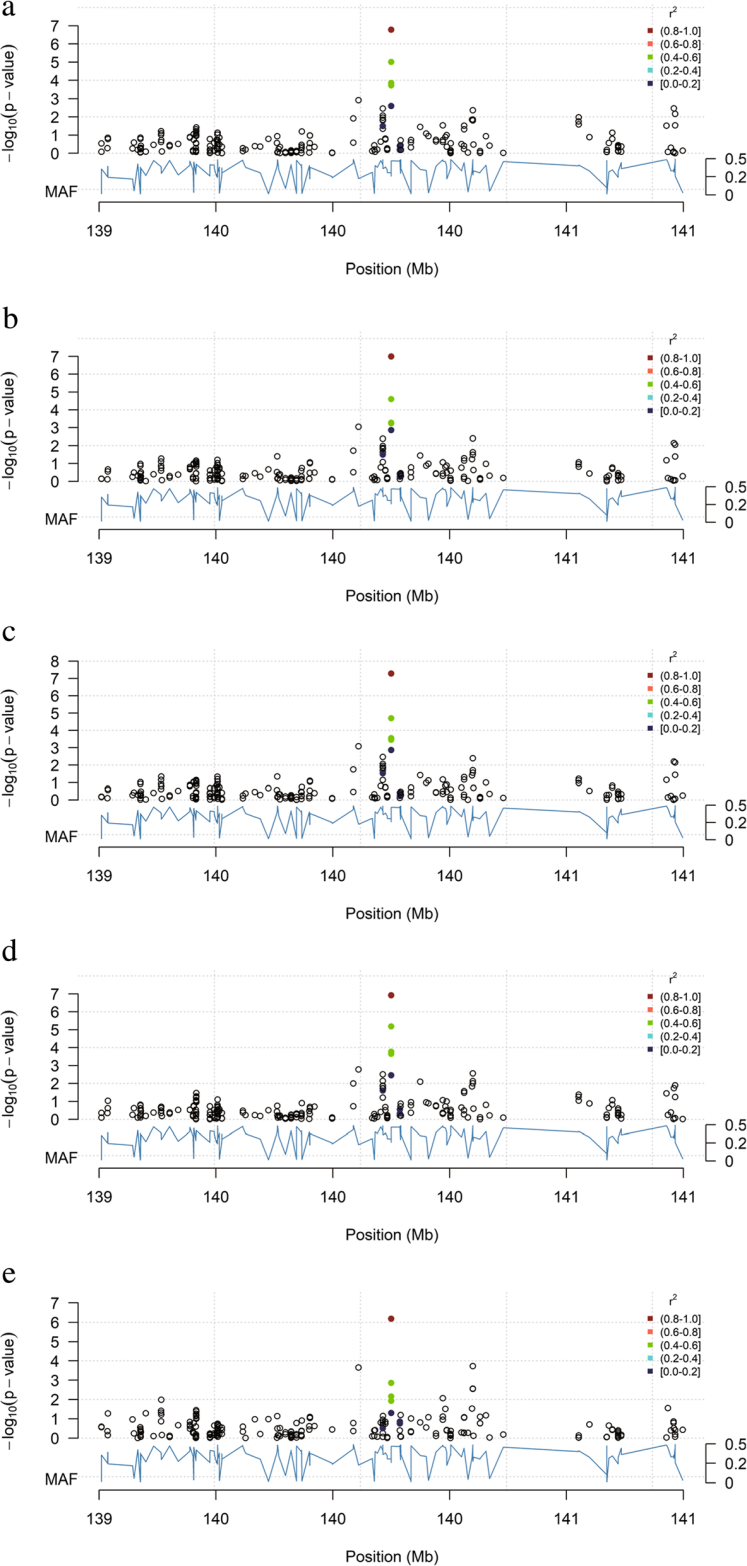
Region of significant loci for DW (**a**), EW (**b**), HEW (**c**), BW42 (**d**), and WW (**e**). Blue curve represents the minor allele frequency; dot color represents the linkage coefficient between the most significant locus and other loci (red highest)

We identified 29 non-overlapping QTLs related to carcass traits. The traits DW, EW, HEW, BW42, and WW shared one genome-wide significant QTL (SNP Chr1_140105435 A > T) (Fig. 2), located in the intron between the last two exons of *ATP11A*. This QTL was also significantly (*P* = 5.27E− 08) associated with HEW, accounting for 5.4% of the genetic variance. There were two significant QTL for FW (Chr1_148339868 A > G, *P* = 7.73E-06; Chr4_59568289 A > G, *P* = 1.62E-05), one in the region of the ATP-binding cassette subfamily C member 4 gene (*ABCC4*) and the other near *LDB2*. We identified two significant QTLs (Chr3_11176648 C > T and Chr10_1656050 A > G) for BMW, located near the transmembrane protein 17 gene (*TMEM17*) and *LOC101804888*, respectively. Three significant QTLs for BMWP were identified on Chr1, of which the most significant locus *LOC101803092* accounted for 8.1% of the genetic variance for this trait. No QTL was identified for LMW, and the SNP Chr20_2198171 C > G, located near the autism susceptibility candidate 2 gene (*AUTS2*), was the only potentially significant QTL for LMWP.

### Gene-set analysis

We further examined the significance of the candidate genes within the QTL using gene set analysis (Table 4). Among the 18 traits, three candidate genes were found to be significant for the four traits BW42, EW, HEW, and LMWP: *LOC101791418*, tubulin gamma complex-associated protein 3 gene (*TUBGCP3*), and *ATP11A* (*P* < 1E− 05). The start and end positions of the three genes were concentrated within the genomic region between 139 and 140 Mb on Chr1. The highest significance was seen for ATP11A with HEW (*P* = 3.83E− 07). These results were consistent with the results of the association analysis above.

**Table 4:** *P*-value of candidate genes in result of gene-set analysis

Gene (Position)	BW42	EW	HEW	LMWP
*ATP11A* (chr1:139901369–140,226,060)	5.05E-06	4.29E-06	3.83E-07	6.50E-06
*LOC101791418* (chr1:140013382–140,221,357)	4.33E-06	1.30E-06	1.90E-06	5.92E-06
*TUBGCP3* (chr1:140058527–140,304,184)	4.93E-06	1.61E-06	2.16E-06	6.75E-06

42-day body weight (BW42), Eviscerated weight (EW), Half-eviscerated weight (HEW) and Leg muscle weight percentage (LMWP)

### Functional annotation of significant regions

A total of 36 candidate genes were detected by GWAS analysis. We performed QTL annotation of these 36 chicken ortholog genes using the chicken QTL database [14] (Additional file 1: Figure S3). Twenty genes were annotated with QTL information in chickens. The QTL information for seven genes was consistent with the carcass traits measured in this study. *ATP11A*, calcium voltage-gated channel subunit alpha1 C (*CACNA1C*), which were associated with 42-day weight-related QTL in chicken studies, were also related to DW, EW, and HEW in ducks in the current study, respectively. Genes *TMEM17* and ATPase plasma membrane Ca^2+^ transporting 1 (*ATP2B1*), as candidate genes associated with BMW and BMWP, were associated with breast muscle weight/percentage QTL in chicken studies. We also annotated the candidate genes using the GO and KEGG databases (Additional file 2: Tables S1 and S2). A total of 25 genes were annotated. Clustering analysis showed that these genes are not significantly enriched in any specific function or biological pathway. The gap junction protein alpha 1 gene (*GJA1*) plays an important role in gap junctions, which are essential for many physiological events, including embryonic development, electrical coupling, metabolic transport, apoptosis, and tissue homeostasis. *ATP2B1* is associated with the calcium signaling pathway and adrenergic signaling in cardiomyocytes. *CACNA1C* was found to be associated with seven pathways, including the calcium signaling pathway, gonadotropin-releasing hormone signaling pathway, and regulation of the actin cytoskeleton.

## Discussion

### Heritability and correlation coefficients

The results of the current study indicated that body size and carcass traits demonstrated moderate to high heritability in Pekin ducks (0.30–0.91). Xu [16] previously estimated that breast muscle weight (BMW) and breast muscle weight percentage (BMWP) had moderate heritabilities (0.23 and 0.16, respectively), while the heritability of body weight was high (0.48). Furthermore, the heritability of BW42 in broilers in Nunes’ study was 0.31 [17]. Compared with these results, we found similar heritabilities of most traits, other than BMW. In this study, we constructed a kinship matrix using SNPs and estimated the heritability of BMW as 0.63, which was obviously higher than Xu’s results. This apparent discrepancy may have been due to population differences or differences in the estimation methods. However, estimations derived from a constructed pedigree from genomic information are likely to be more accurate than those from traditional pedigree records as the genomic information reflect more real genetic relationship than traditional pedigree.

The largest phenotypic correlations were between DW and EW and between DW and HEW, while the genetic correlation between BW42 and DP was small (0.39). Nunes et al. [17] estimated the genetic parameters related to body weight, chemical carcass composition, and yield in a broiler-layer cross and found a genetic correlation between BW42 and DP of 0.33, which was in accord with the current results. We found highly positive genetic (0.74) and phenotypic correlations (0.63) between BW42 and BMW. Venturini et al. [18] also found genetic and phenotypic correlation coefficients of 0.86 and 0.84 between BW42 and BMW, respectively, which were consistent with our results. However, compared with Venturini et al.’s result (0.7), we found a smaller genetic correlation between BW42 and LMW (0.3), likely due to the use of a different species. Overall, our results indicated that the genetic parameters related to body weight traits in the Pekin duck population were consistent with previous studies.

### Candidate genes related to body size, body weight and carcass traits

In this study, *ATP11A* was significant in both loci-based model GWAS and gene-set GWAS, and was associated with DW, EW, HEW, BW42, WW, and LMWP. Based on chicken QTL information, *ATP11A* was also associated with body weight and internal organ weight in chickens. Also, Segawa et al. [19] noted that the major flippases at the plasma membrane in most mammal cells are encoded by *ATP11A* and *ATP11C*. *ATP11A* encodes a complete membrane ATPase involved in the transport of phospholipids. Transporting creates membrane phospholipid asymmetry and initiates the biogenesis of transport vesicles. We hypothesize that this gene may be involved in metabolism in duck fat cells, and may be highly significantly related to five traits because body weight is a complex trait that involves the development of multiple organs. This gene may thus be involved in basic growth and development processes, especially in relation to fat deposition; however, more experiments are needed.

The measurement of carcass traits in poultry breeding is both expensive and difficult, and can only be conducted after death. In contrast, BrW is an important live body measurement that is closely related to body weight and breast muscle production [20]. The current results revealed very high genetic and phenotypic correlation coefficients between BrW and EW, HEW, and BW42, respectively. All of them share the same SNP (Chr2_19666206 A > G). It was one of the most significant SNPs for BrW, accounting for 9.8% of the observed genetic variance, with a MAF of 0.479. The candidate gene was *PLXDC2*, which coordinates the development and differentiation of nerve cells in various animals, including humans, mice, and chickens [21–23]. Our results demonstrated correlations between this site and BW42, DW, EW, HEW, and BrW, and chicken QTL annotation showed that the gene was associated with both body weight and leg muscle weight.

Like BrW, FBL can also be measured easily and is significantly correlated with weight traits, and can thus be used for trait selection indirectly. The results of the present study showed that all the genetic and phenotypic correlation coefficients between FBL and BW42, DW, EW, HEW, and WW, respectively, were high. We also found that the SNP Chr2_52398515_C > T (located in intron of *TNS3*), which was significantly associated with FBL, accounted for 6.2% of the genetic variance. The *TNS* gene family plays an important role in the development and formation of bones. Dedicator of cytokinesis 5 (*Dock5*) and *TNS3* genes have been shown to have a synergistic effect on the maintenance of osteoclast activity to ensure the correct organization of podosomes [24]. QTL information indicated that the QTL related to body weight and breast muscle weight/percentage localized to *TNS3*. However, the trait of FBL was not included in the chicken QTL information and we were unable to find any previous reports of FBL in poultry. We therefore provide the first report of a QTL associated with FBL in ducks, as an important reference for future research.

Secondary duck products after slaughter represent a large market share in Asia, and a better understanding of the genetic bases of the relevant economic traits will help to increase yield. In our study, the candidate gene for FW was *LDB2*, which encodes a LIM-domain-binding family protein that binds to a variety of transcription factors and plays a crucial role in brain development and angiogenesis [25, 26]. Wang et al. [27] conducted a GWAS in 400 chickens from a conservation population of a local Chinese breed (Jinghai Yellow chickens). They identified five SNPs with genome-wide significance for FW, including one for which the candidate gene was *LDB2*, located on Chr4, as in the current study. Gu et al. [8] also found that the SNP within *LDB2* had the strongest association with late growth (body weight from 7 to 12 weeks old and average daily weight gain from 6 to 12 weeks old).

The current study identified four suggestive sites for HW, distributed on four different chromosomes. The candidate gene for one of these, *GJA1*, encodes connexin43 and connexin45, which comprise part of the gap junction consisting of an array of intercellular channels that provides a pathway for the diffusion of low-molecular-weight substances between cells [28]. Proteins are thought to play a key role in the synchronous contraction of the heart and in embryonic development [29]. Recent studies have also demonstrated that *GJA1* is an important functional gene in chicken growth and development, especially of chicken breast muscle [7, 30]. Other functions of this gene have yet to be explored; however, the high correlation between BMW and HW suggests that *GJA1* might be an important gene in terms of increasing breast muscle yield.

We also identified genes such as that encoding Kruppel-like factor 5 (*KLF5*) in relation to DW, EW, HEW, and HW, and *ATP2B1* for BMWP. KLFs are zinc-finger transcription factors that act as key regulators of cellular differentiation and growth in adipocytes [31]. *KLF5* is involved in the biological process that regulates lipid storage, and thus affects body weight-related traits. However, further studies are needed to clarify the role of *ATP2B1* in poultry.

The use of molecular markers, revealing polymorphism at the DNA level, has been playing an increasing part in animal genetics studies. If we obtained more QTLs for duck, it would be possible to integrate these SNPs information in the genomic selection scheme to improve the selection accuracy in ducks.

## Conclusions

In this study, we conducted a GWAS of 18 carcass traits in Pekin ducks. We detected 37 QTLs distributed across 26 chromosomes, and identified 36 candidate genes related to body size and carcass traits. These findings further our understanding of poultry genetics and provide a genetic basis for breeding programs aimed at maximizing the economic potential of Pekin ducks.

## Methods

### Ducks and phenotypes

A total of 639 (males: 314; females: 325) 21-day-old Pekin ducks from the same flock were randomly selected at Beijing Golden Duck Co., Ltd. (Beijing, China). They were divided randomly into five batches (120, 114, 117, 134, and 154 ducks each). The interval between each batch was 5 days. All ducks were fed with the same diet and maintained under the same lighting condition, as described previously [32]. Ducks were weighed at 21 and 42 days old. After fasting for 6 h, all the ducks were slaughtered at d 42 according to standard commercial procedures. Euthanasia was performed by cervical dislocation. A total of 18 traits were measured according to the performance terms and measurement of poultry guidelines issued by the Ministry of Agriculture of China (NY/T 823–2004), including neck length (NL), fossil bone length (FBL), breast width (BrW), dressed weight (DW), dressed percentage (DP), eviscerated weight (EW), eviscerated weight percentage (EWP), half-eviscerated weight (HEW), percentage of half-eviscerated yield (HEWP), 42-day body weight (BW42), heart weight (HW), liver weight (LW), foot weight (FW), wing weight (WW), breast muscle weight (BMW), breast muscle weight percentage (BMWP), leg muscle weight (LMW), and leg muscle weight percentage (LMWP) [32]. The following traits were measured using a caliper and weighing scale and calculated as follows:

Neck length: the distance between the first cervical vertebra and the end of neck;

Fossil bone length: The distance between the anterior and the posterior border of the breast-bone crest;

Breast width: the distance vertically between the backbone and the beginning of the breast-bone crest;

Dressed weight: Weight after bloodletting and removal of feathers, foot cuticles, toes, and clam shells;

Dressed percentage (%) = Dressed weight/slaughter weight × 100;

Half-eviscerated weight: Weight of carcass after removal of trachea, esophagus, crop, intestines, spleen, pancreas, gallbladder, reproductive organs, stomach contents, and keratinocytes;

Percentage of half-eviscerated yield (%) = Half-eviscerated weight/slaughter weight × 100;

Eviscerated weight: Half-eviscerated weight minus the weight of the heart, liver, stomach, lungs, and abdominal fat;

Percentage of eviscerated yield (%) = Eviscerated weight/slaughter weight × 100;

Slaughter weight/42-day body weight: Weight after fasting for 6 h;

Percentage of breast muscle (%) = Breast muscle weight/eviscerated weight × 100;

Leg muscle: Total leg muscle weight after removing leg bones, skin, and subcutaneous fat;

Leg muscle weight percentage of (%) = Leg muscle weight/eviscerated weight × 100.

### GBS

Fresh blood was collected from the ducks before slaughtering and genomic DNA was extracted from the blood using a phenol–chloroform-based method. Genotyping was performed using GBS, as described previously [15]. A total of 544 million clean reads (63.25 GB) were generated, of which 96.12% (523 million reads) were mapped to the duck genome, with an average mapping rate of 96.25%. The data were deposited in the NCBI sequence read archive (SRP068685). GBS procedures are available on Zhu Feng’s article [15]. Restriction enzyme (*MSe1*) for the PCR-RFLP assay was selected using information from REBASE [33]. A set of variable barcode adapters that recognize Mse1-compatible sequences were ligated to the digested DNA fragments. The restriction fragments were enriched by PCR amplification with adapter-specifc primers [34]. Te data of 2 × 125 bp pair-end reads were generated by the Illumina HiSeq2500.

### Single nucleotide polymorphism (SNP) identification

Clean data were mapped to a reference genome using BWA (version 1.73) [35]. The reference genome was a chromosome-assembly version from BGI duck 1.0 reference (GCA_000355885.1) based on the RH map [36] with ALLMAP [37]. VCFtools [38] and PLINK (1.90) [39] were used for quality control of the data. SNP detection was performed using the GATK HaploCaller (3.7) [40]. All parameters were kept at default settings, except for -stand_call_conf 30. The data were imputed using Beagle (4.1) [41], using R^2^ > 0.3 for low-quality filtering. A total of 62,067 SNPs met one or more of the following conditions: minor allele frequency (MAF) of > 1%, sample call rate ≥ 95%, and the Hardy–Weinberg equilibrium test (*P* > 10^− 6^). All phenotype measurements were normalized using the rank transformation method, and the effects of batch and gender on the phenotype were examined using the variance test for subsequent covariates of the mixed model. Population substructure analysis was performed using EIGENSOFT (2.04), and the top ten PCA [42] components were used as covariates for further analysis. GCTA-LDMC [43] was used to estimate the genetic parameters. SNPs used in this study listed in Additional file 2: Table S3.

### Statistical analysis

Loci-based analysis was performed using the generalized linear mixed model implemented in GEMMA [44], where the kinship matrix was calculated using the center method. The mixed model was mainly based on the additive effect of sites:

$$ y=1\mu + Xb+u+ Sa+e $$

where y is the vector phenotypes of per duck; μ is the overall mean; X is the covariance matrix (mainly containing the gender effect, batch effect, and first ten PCA principal components obtained from the analysis of population substructure); b is the estimator vector of fixed effects; u ~ (0, G${\upsigma}_{\mathrm{u}}^2$) is the additive polygenic effect, with G the genomic kinship matrix, and ${\upsigma}_{\mathrm{u}}^2$ is the additive effect variance; S is the design matrix containing the corresponding SNP sites; α is the substitution effect size corresponding to each site; and e~N (0, I${\upsigma}_{\mathrm{e}}^2$) is the vector of random residual effects, with I the identity matrix and ${\upsigma}_{\mathrm{e}}^2$ the residual variance.

Gene-set analysis used the MAGMA Top model [45]. Gene analysis in MAGMA is based on a multiple linear principal components regression model, using an F-test to compute the gene *P*-value. The association level for each gene was the weighted sum of the associated statistics for the SNP sites in the region. Using this model can improve the statistical power for the identification of a candidate gene. To perform the gene-set analysis, for each gene g the gene *p*-value *p*_*g*_ computed with the gene analysis is converted to a Z-value *z*_*g*_ = Φ^− 1^(1–*p*_*g*_), whereΦ^− 1^ is the probit function. This yields a roughly normally distributed variable Z with elements z g that reflects the strength of the association each gene has with the phenotype, with higher values corresponding to stronger associations.

The genomic inflation factor (λ) was calculated using R package qqman [46]. Multiple test thresholds were calculated using the simpleM method [47]. A total of 28,707 valid inspections were obtained. The genome-wide significance level was 1.741735E− 06 (0.05/28,707), and the suggestive significance level was 3.483471E− 05 (1/28,707). LD statistic were performed using PLINK (−-r2) to calculates inter-variant allele count correlations [39]. Multiple consecutive significant sites were defined as separate QTL regions, and site-effect values were calculated using the equation 2pqβ^2^/σ^2^, with allele frequencies p and q, β is corresponding effect size of SNP identified in association study, σ^2^ is phenotypic variance.

### Functional annotation

The gene position information was annotated using BEDTools [48]. The functions of the genes were annotated using the KEGG and GO databases, and enrichment analysis was performed using the R package GOSeq [49]. Due to the absence of a duck QTL database, we used the orthologues of chicken genes and their QTL information for candidate genes in the Animal QTL Database [14].

## Additional files

Additional file 1: **Figure S1** Manhattan plots showing associations of all SNPs with all traits (a. NL, b. FBL, c. BrW, d. DW, e. DP, f. EW, g. EWP, h. HEW, i. HEWP, j. BW42, k. HW, l. LW, m. FW, n. WW, o. BMW, p. BMWP, q. LMW, r. LMWP). SNPs are plotted on the x-axis according to their position on each chromosome, against association with these traits on the y-axis (shown as −log10p-value). Solid blue line indicates suggestive significance association (*P* = 3.48E-05) and red solid line shows genome-wide significance with a *P*-value threshold of 1.74E-06. **Figure S2** Quantile-quantile (Q-Q) plots of the GLM (black dots) for carcass traits (a. NL, b. FBL, c. BrW, d. DW, e. DP, f. EW, g. EWP, h. HEW, i. HEWP, j. BW42, k. HW, l. LW, m. FW, n. WW, o. BMW, p. BMWP, q. LMW, r. LMWP). Expected *P*-values under the null hypothesis are plotted on the x-axis and observed *P*-values on the y-axis. **Figure S3** QTL information for ortholog chicken candidate genes. There is no duck QTL database and we therefore used chicken QTL information for the candidate genes in the Animal QTL Database. This figure was based on the shared QTL between our study in ducks and the chicken QTL database. The color intensity represents the degree of enrichment of similar QTL. Liver weight in ducks and carcass proportion in chickens shared the most QTL (9). Foot weight, wing weight, and breast muscle weight in ducks shared no QTL with body size in chickens. (DOCX 855 kb)

Additional file 2: **Table S1** GO analysis of the candidate genes. **Table S2** Biological pathways of the candidate genes. **Table S3** All SNP information. (XLSX 1050 kb)

## Abbreviations

BMW: Breast muscle weight
BMWP: Breast muscle weight percentage
BrW: Breast width
BW42: 42-day body weight
DP: Dressed percentage
DW: Dressed weight
EW: Eviscerated weight
EWP: Eviscerated weight percentage
FAO: Food and Agriculture Organization
FBL: Fossil bone length
FW: Foot weight
GBS: Genotyping-by-Sequencing
GO: Gene ontology
GWAS: Genome-wide association study
HEW: Half-eviscerated weight
HEWP: Percentage of half-eviscerated yield
HW: Heart weight
KEGG: Kyoto encyclopedia of genes and genomes
LD: Linkage disequilibrium
LMW: Leg muscle weight
LMWP: Leg muscle weight percentage
LW: Liver weight
NL: Neck length
PCA: Principal component analysis
QTL: Quantitative trait loci
SNP: Single nucleotide polymorphism
WW: Wing weight
