# Supplementary figures and images for: Genome-wide association study reveals novel loci associated with body size and carcass yields in Pekin ducks
Source: BMC Genomics. 2019 Jan 3;20:1. doi: 10.1186/s12864-018-5379-1 (PMC6318962; doi:10.1186/s12864-018-5379-1)

Additional file 1:

Figure S1


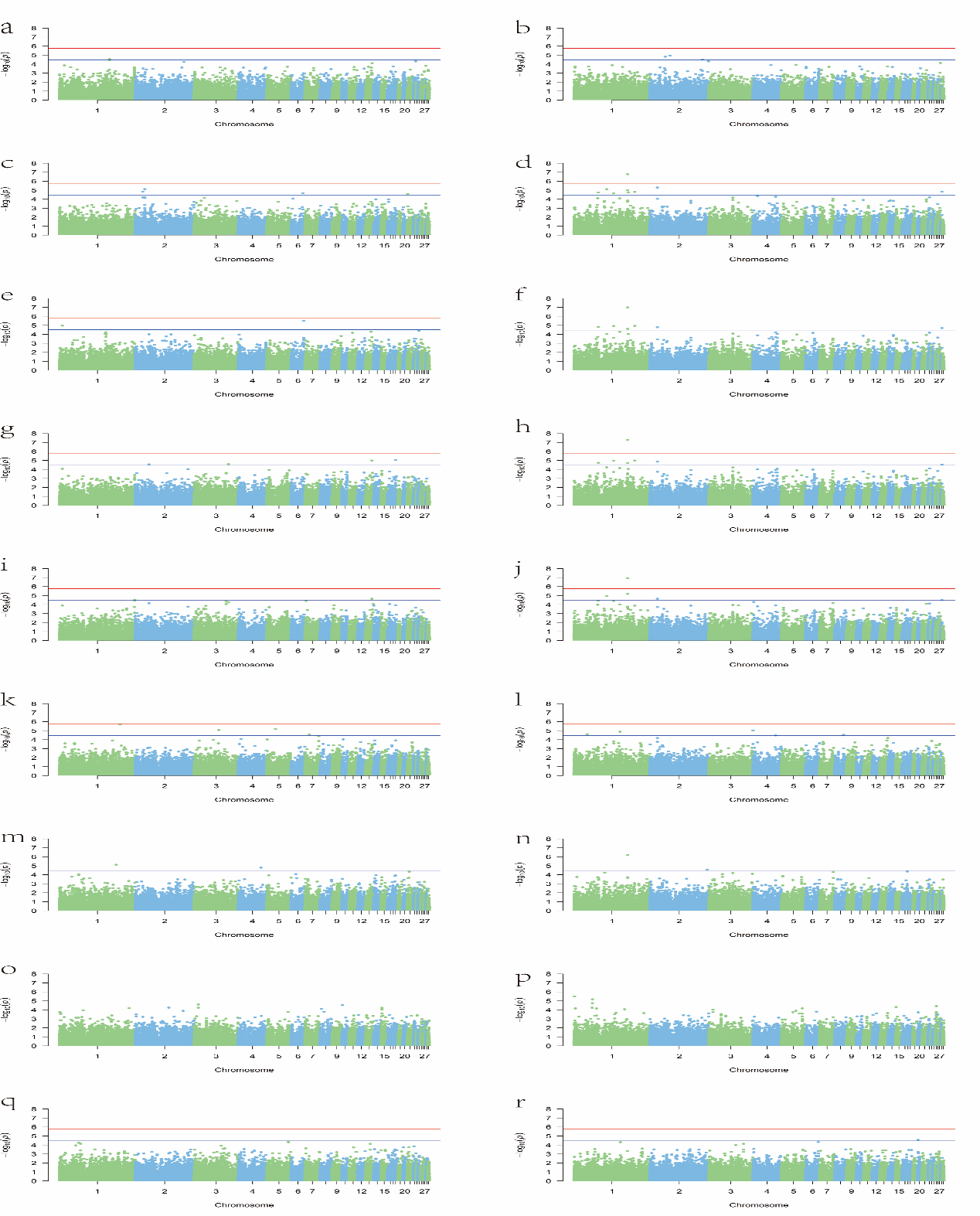


Figure S2


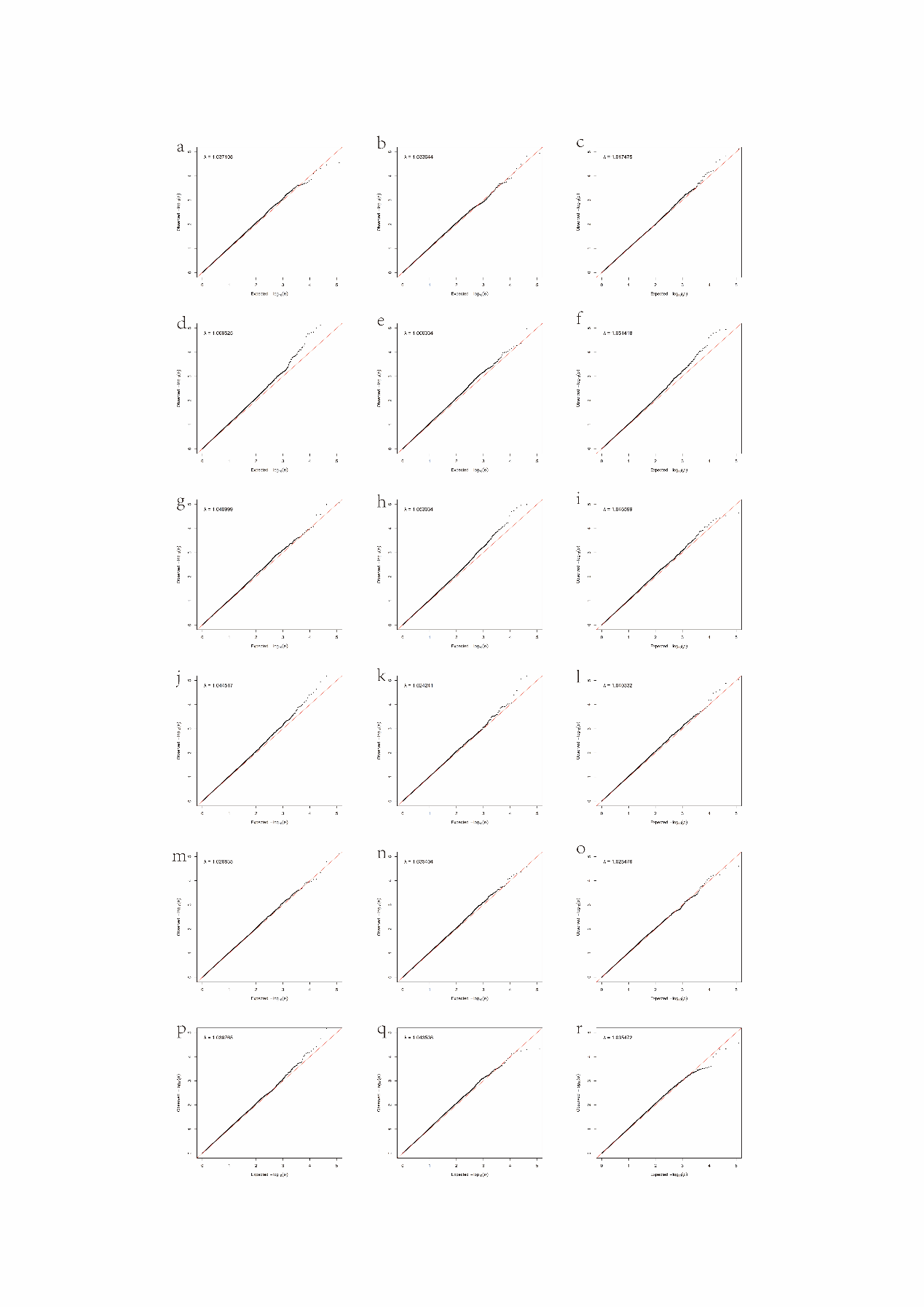


Figure S3


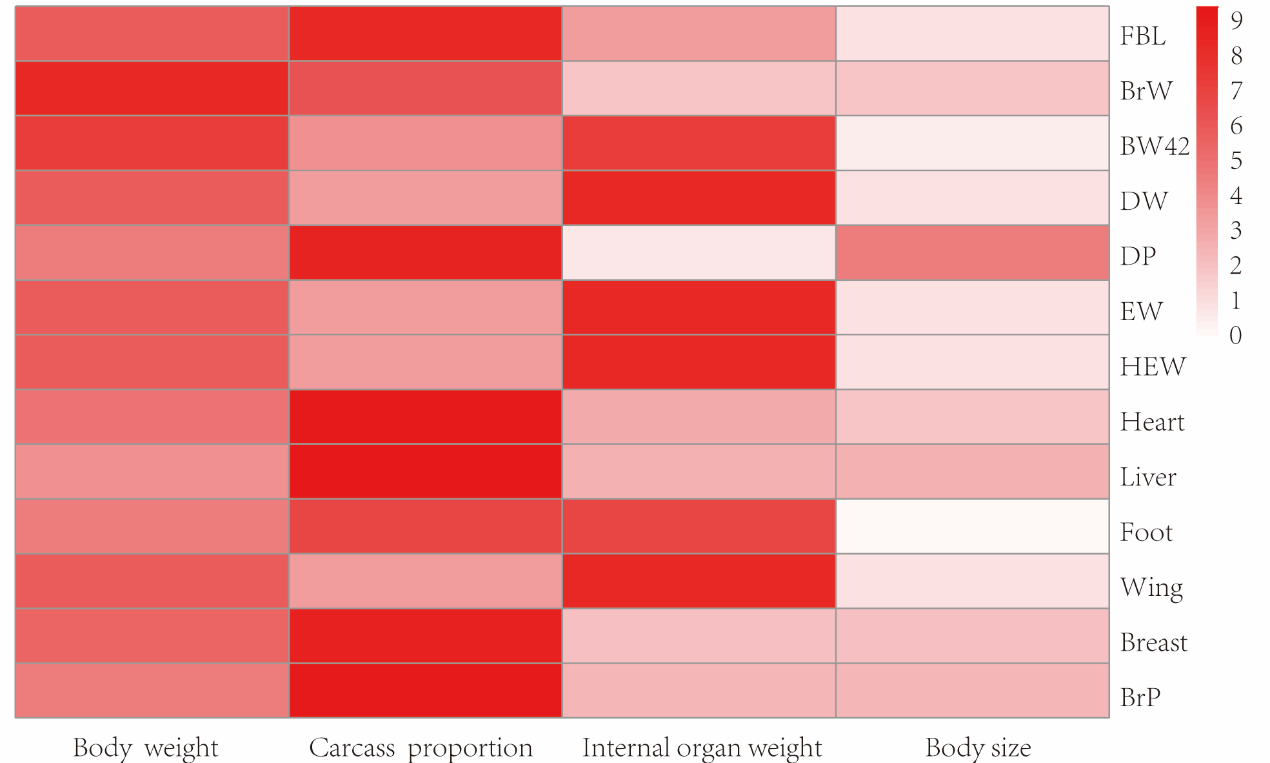

Supplement: Supplementary file 1 — Figure S1 Manhattan plots showing associations of all SNPs with all traits (a. NL, b. FBL, c. BrW, d. DW, e. DP, f. EW, g. EWP, h. HEW, i. HEWP, j. BW42, k. HW, l. LW, m. FW, n. WW, o. BMW, p. BMWP, q. LMW, r. LMWP). SNPs are plotted on the x-axis according to their position on each chromosome, against association with these traits on the y-axis (shown as −log10p-value). Solid blue line indicates suggestive significance association (P = 3.48E-05) and red solid line shows genome-wide significance with a P-value threshold of 1.74E-06. Figure S2 Quantile-quantile (Q-Q) plots of the GLM (black dots) for carcass traits (a. NL, b. FBL, c. BrW, d. DW, e. DP, f. EW, g. EWP, h. HEW, i. HEWP, j. BW42, k. HW, l. LW, m. FW, n. WW, o. BMW, p. BMWP, q. LMW, r. LMWP). Expected P-values under the null hypothesis are plotted on the x-axis and observed P-values on the y-axis. Figure S3 QTL information for ortholog chicken candidate genes. There is no duck QTL database and we therefore used chicken QTL information for the candidate genes in the Animal QTL Database. This figure was based on the shared QTL between our study in ducks and the chicken QTL database. The color intensity represents the degree of enrichment of similar QTL. Liver weight in ducks and carcass proportion in chickens shared the most QTL (9). Foot weight, wing weight, and breast muscle weight in ducks shared no QTL with body size in chickens. (DOCX 855 kb) [file 12864_2018_5379_MOESM1_ESM.docx]
